# Acute echocardiographic and electrocardiographic effects of triggered left ventricular pacing

**DOI:** 10.1371/journal.pone.0278531

**Published:** 2022-12-06

**Authors:** Tobias Jonathan Pfeffer, Jonas Neuser, Johanna Mueller-Leisse, Stephan Hohmann, David Duncker, Johann Bauersachs, Christian Veltmann, Dominik Berliner

**Affiliations:** 1 Department of Cardiology and Angiology, Hannover Medical School, Hannover, Germany; 2 Electrophysiology Bremen, Bremen, Germany; University Medical Center Groningen, University of Groningen, NETHERLANDS

## Abstract

Cardiac resynchronization therapy (CRT) is an essential pillar in the therapy of heart failure patients with reduced ejection fraction (HFrEF) presenting with broad left bundle branch block (LBBB) or pacemaker dependency. To achieve beneficial effects, CRT requires high bi-ventricular (BiV) pacing rates. Therefore, device-manufacturers designed pacing algorithms which maintain high BiV pacing rates by a left ventricular (LV) pacing stimulus immediately following a right ventricular sensed beat. However, data on clinical impact of these algorithms are sparse. We studied 17 patients implanted with a CRT device providing triggered left ventricular pacing (tLVp) in case of atrioventricular nodal conduction. Assessment of LV dyssynchrony was performed using echocardiographic and electrocardiographic examination while CRT-devices were set to three different settings: 1. Optimized bi-ventricular-stimulation (BiV); 2. Physiological AV nodal conduction (tLVp-off); 3. Physiological AV nodal conduction and tLVp-algorithm turned on (tLVp-on). QRS duration increased when the CRT-device was set to tLVp-off compared to BiV-Stim, while QRS duration was comparable to BiV-Stim with the tLVp-on setting. Echocardiographic analysis revealed higher dyssynchrony during tLVp-off compared to BiV-Stim. TLVp-on did not improve LV dyssynchrony compared to tLVp-off. QRS duration significantly decreased using tLVp-algorithms compared to physiological AV nodal conduction. However, echocardiographic examination could not show functional benefit from tLVp-algorithms, suggesting that these algorithms are inferior to regular biventricular pacing regarding cardiac resynchronization. Therefore, medical treatment and ablation procedures should be preferred, when biventricular pacing rates have to be increased. TLVp-algorithms can be used in addition to these treatment options.

## Introduction

Cardiac resynchronization therapy (CRT) has become an essential pillar in the therapy of patients suffering from heart failure with reduced ejection fraction (HFrEF) presenting with broad left bundle branch block (LBBB) or pacemaker dependency [1, 2]. Numerous reports have shown the benefit of CRT with respect to morbidity and mortality [3–7]. However, several studies revealed a necessity for high bi-ventricular (BiV) pacing rates to achieve these beneficial effects [8–11]. In clinical practice, reaching high BiV pacing rates might be challenging due to rapid intrinsic atrioventricular conduction during atrial tachycardia or atrial fibrillation [12–14]. Furthermore, premature ventricular contractions (PVC) can lead to a significant reduction in BiV pacing rates [15, 16]. In order to maintain high BiV pacing rates in the presence of atrioventricular (AV) nodal conduction, CRT-device manufacturer designed algorithms such as Ventricular Sense Response (Medtronic), RVsense (Biotronik), BiV trigger (Boston Scientific) or ventricular triggered pacing (St. Jude Medical/Abbott) to ensure cardiac resynchronization by pacing the left ventricle (LV) immediately after a right ventricular (RV) event has been sensed [17].

However, even if the approach of increasing the BiV pacing rate by using these algorithms is mechanistically conclusive, in contrast to BiV pacing and LV univentricular pacing data regarding the functional effects of triggered left ventricular pacing (tLVp) following right ventricular sensing are sparse [18–22]. We therefore sought to investigate the impact of tLVp-algorithms in patients suffering from HFrEF with regard to echocardiographic and electrocardiographic parameters of cardiac synchronization.

## Materials and methods

The study procedures were in accordance with the ethical guidelines of the 1975 declaration of Helsinki and the local ethic committee of the Hannover Medical School approved the study protocol (ethics application number: 9865_BO_K_2021). All patients agreed to the inclusion in this study and provided written and informed consent to the scientific analysis of their data.

### Patient population

We screened patients referred to our device outpatient clinic for routine device follow-up, suffering from HFrEF and LBBB, defined by QRS duration ≥ 140 ms for men and ≥ 130 ms for women and mid-QRS notching or slurring in ≥ 2 contiguous leads according to the definition by Strauss et al. [23], implanted with a CRT-device providing a tLVp-algorithm. Patients with underlying heart rate ≥ 45 bpm, intrinsic atrioventricular nodal conduction, LBBB, sufficient echocardiographic acoustic window and stable lead measurements were included.

### CRT stimulation settings

**Fig 1:**
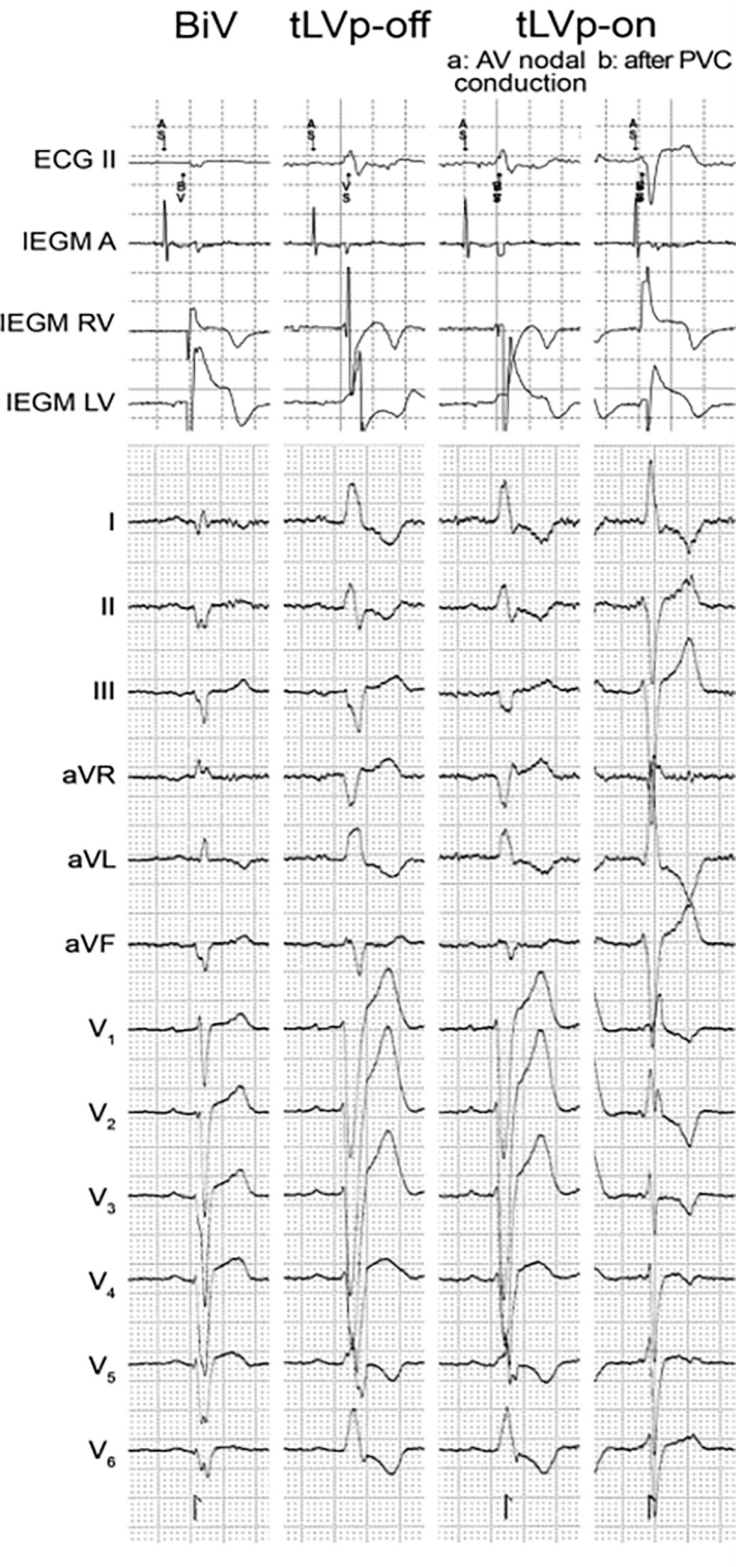
Electrogram (EGM) and electrocardiogram (ECG) of the three different settings: 1. optimized bi-ventricular-stimulation (BiV); 2. Physiological AV nodal conduction without triggered left ventricular pacing (tLVp-off); 3. TLVp algorithm turned on (tLVp-on) with physiological AV nodal conduction (a), and after premature ventricular contraction (PVC; b). (ECG–electrocardiogram, IEGM–internal electrogram, PVC—premature ventricular contraction, tLVp—triggered left ventricular pacing).

Captures of echocardiographic views and record of an electrocardiogram was done, while CRT devices were set to three different programming modes: First, CRT device set to a patient specific optimized BiV-pacing setting (BiV). Secondly, we changed the mode to a VVI 40 bpm allowing permanent intrinsic atrioventricular nodal conduction (tLVp-off). Then the CRT device was set to the same VVI backup mode with the tLVp-algorithms turned on (tLVp-on), leading to a permanent LV pacing stimulus following the right ventricular sensed event (Fig 1).

### Electrocardiographic and echocardiographic examination

Electrocardiogram (ECG) recording and echocardiographic examination were done after an equilibrating-phase of 5 minutes. A standard ECG workstation (Mac5000, GE healthcare, Chicago, USA) was used to record a 12-lead ECG. Transthoracic echocardiography was performed utilizing a Philips EPIQ 7c ultrasound machine (Philips, Amsterdam, Netherlands). Standard apical 4-, 2-, and 3-chamber views as well as a 3-dimensional full volume were acquired capturing five respectively four heart cycles during each stimulation mode. Off-line analysis of 2-dimenional (2D) longitudinal strain (LS) and 3D volumetry was done using TOMTEC cardiac measurement software (TomTec Imaging Systems, Unterschleissheim, Germany). Two parameters concerning synchrony were assessed: Using the 3D full volume, the dyssynchrony index (SDI) was determined. The SDI is based on the 16-segment model and derives from standard deviation of the regional end-systolic times [24]. Additionally, we analyzed the delay of two opposite wall segments in 2D LS. Maximum delay was defined as the maximum delay between opposite wall segments derived from 4-, 2-, and 3-chamber views.

### Statistical analysis

Statistical analyses of the clinical data were performed using IBM SPSS 26 (IBM, Armonk, New York, USA). Continuous data are expressed as median with interquartile range. Categorical variables are presented as frequencies (percentages). Comparison between the different CRT stimulation modes were performed using a two-way analysis of variance by ranks (Friedman’s test). A pairwise Dunn test with Bonferroni correction was performed as post-hoc test for the Friedman test. All p-values are two-sided and a p-value of <0.05 was considered as statistically significant. In order to establish a prediction model, which patients might benefit from tLVp-algorithms, we performed subgroup analysis between patients with good electrocardiographic CRT response and patients with poor electrocardiographic CRT response. Electrocardiographic CRT response was defined by the difference of the QRS duration between intrinsic atrioventricular nodal conduction, while the tLVp-algorithm was switched off, and tLVp. A difference above the median was classified as full response whereas a difference of below the median was classified as poor response.

## Results

### Patient characteristics

Patients’ characteristics are shown in Table 1. In brief, a total of 17 patients with a mean age of 68 years [interquartile range (IQR) 53; 74] were included. Patients were primarily male (88%) and ischemic heart disease (47%) and dilative cardiomyopathy (47%) were the most common etiologies of LV dysfunction; one patient had non-compaction cardiomyopathy. All patients presented with a broad LBBB of at least 130ms with a median QRS duration of 170 [IQR 160; 180]ms. The majority of patients reported a functional capacity evaluated as NYHA class II (88%) under optimal CRT. Pharmacological treatment comprised ACE-inhibitor, AT-1-receptor-blocker or angiotensin receptor-neprilysin inhibitor in all patients and beta-blockers in 94% as well as mineralocorticoid-antagonists in 94% of the patients. Patients were implanted with devices from several manufacturers including St. Jude Medical/Abbott^TM^ (13/17; 77%), Medtronic^TM^ (2/17; 12%), and Biotronik^TM^ (2/17; 12%). In all patients the RV lead was placed in the right apex. LV lead position was analyzed in fluoroscopic RAO and LAO view: The LV leads were mostly located in the posterolateral position (80%), followed by the posterior position (13%), in 1 case in the lateral position, in (60%) in the midventricular segment followed by the apical segment (20%) and the basal segment (20%). In 73% of the patients a quadripolar lead was implanted. Pacing vector was chosen by the treating physician aiming for optimized electrical activation while, a pacing threshold as low as possible and the avoidance of phrenic nerve stimulation. In patients with a quadripolar lead, the LV lead position refers to the selected left ventricular pacing configuration. All patients were classified as CRT responders by the treating physician and BiV pacing rates were >95% in all patients. Median paced AV-interval was 150ms [IQR 140; 160ms], while sensed AV time was 110ms [IQR 100; 120ms]. Median of interventricular interval was 20ms [IQR 0; 40] LV before RV stimulation. In 5.9% the device was equipped with a dynamic acting algorithm optimizing CRT pacing (e.g. AdaptivCRT^TM^ by Medtronic).

**Table 1:** Patient characteristics (n = 17).

Parameters	Patients (n = 17)
Age (years)	68 [53; 74]
Male	88.2%
NYHA functional class	
I	5.9%
II	88.2%
III	5.9%
Cause of left ventricular dysfunction	
Ischemic cardiomyopathy	47.1%
Dilated cardiomyopathy	47.1%
Non-compaction cardiomyopathy	5.9%
3D left ventricular ejection fraction (%)	36.7 [30.4; 38.2]
Left bundle branch block	100%
Intrinsic QRS width (ms)	170 [160; 180]
QRS width (ms)–with biventricular stimulation	130 [120; 150]
Device related	
Manufacturer	
St Jude Medical/AbbottTM	76.5%
MedtronicTM	11.8%
BiotronikTM	11.8%
Percentage of biventricular stimulation (%)	99.0 [99.0; 99.9]
Heart Failure Medication	
Beta-Blocker	94.1%
ACE-I / ARB / ARNI	100%
Mineralocorticoid receptor antagonists	94.1%
Diuretics	88.2%

(3D - 3-dimensional, ACE-I–Angiotensin Converting Enzyme inhibitor; ARB–Angiotensin-1-Receptor Blocker; ARNI–Angiotensin Receptor-Neprilysin Inhibitor (Sacubitril/Valsartan). NYHA–New York Heart Association. Data are given as median [interqartile range] or proportion of all cases).

### Electrocardiographic data

**Fig 2:**
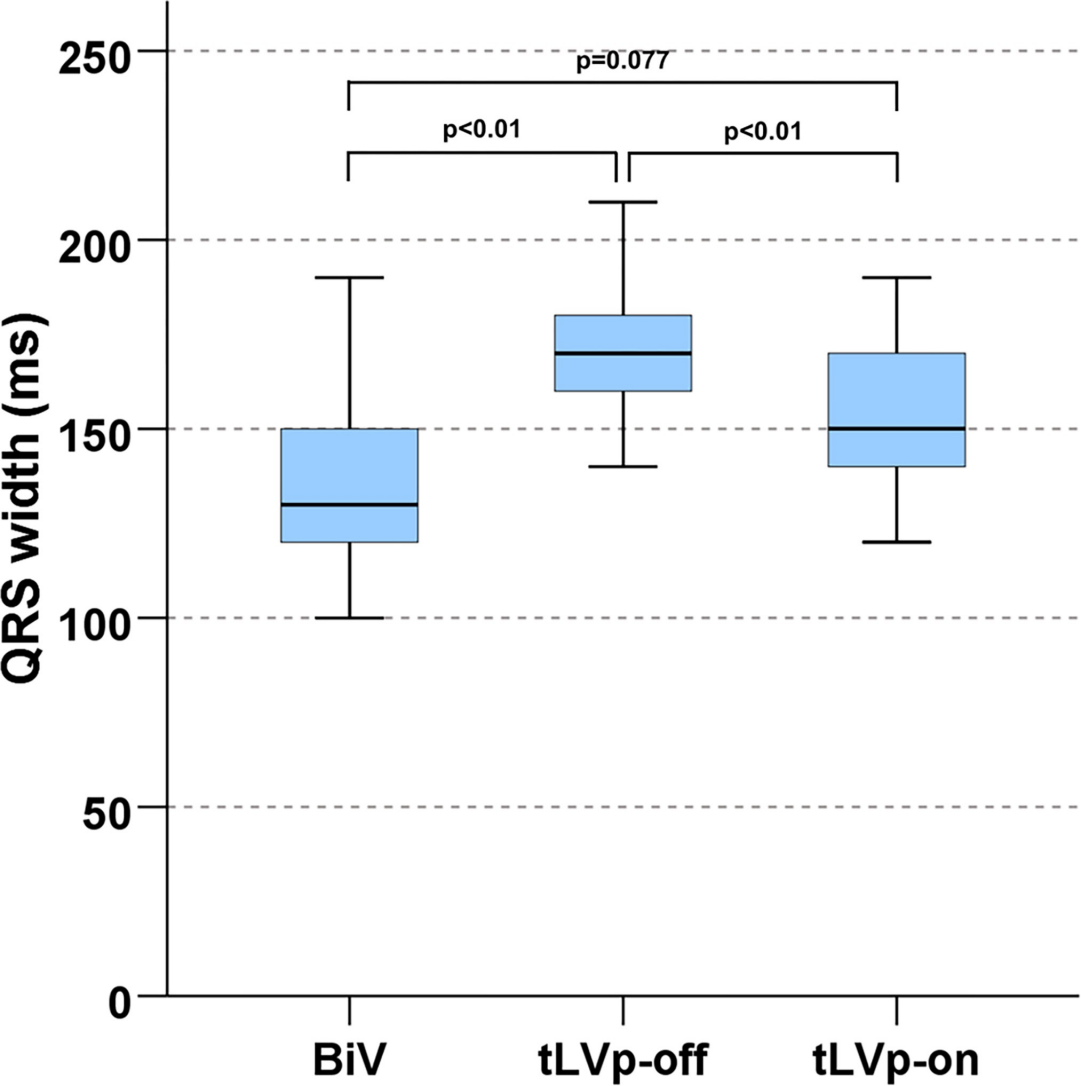
QRS width (ecg) in the different CRT modes. (BiV–biventricular pacing, LV—left ventricle, tLVp—triggered left ventricular pacing).

Compared to BiV-Stim (130 ms [IQR 120; 150 ms]), QRS duration increased significantly under intrinsic AV nodal conduction (170 ms [IQR 160; 180 ms], p<0.01). After programming to the tLVp-on setting, QRS duration in turn significantly decreased (150 ms [IQR 140; 170 ms], p<0.01) compared to the tLVp-off setting (Fig 2).

### Echocardiographic data

**Fig 3:**
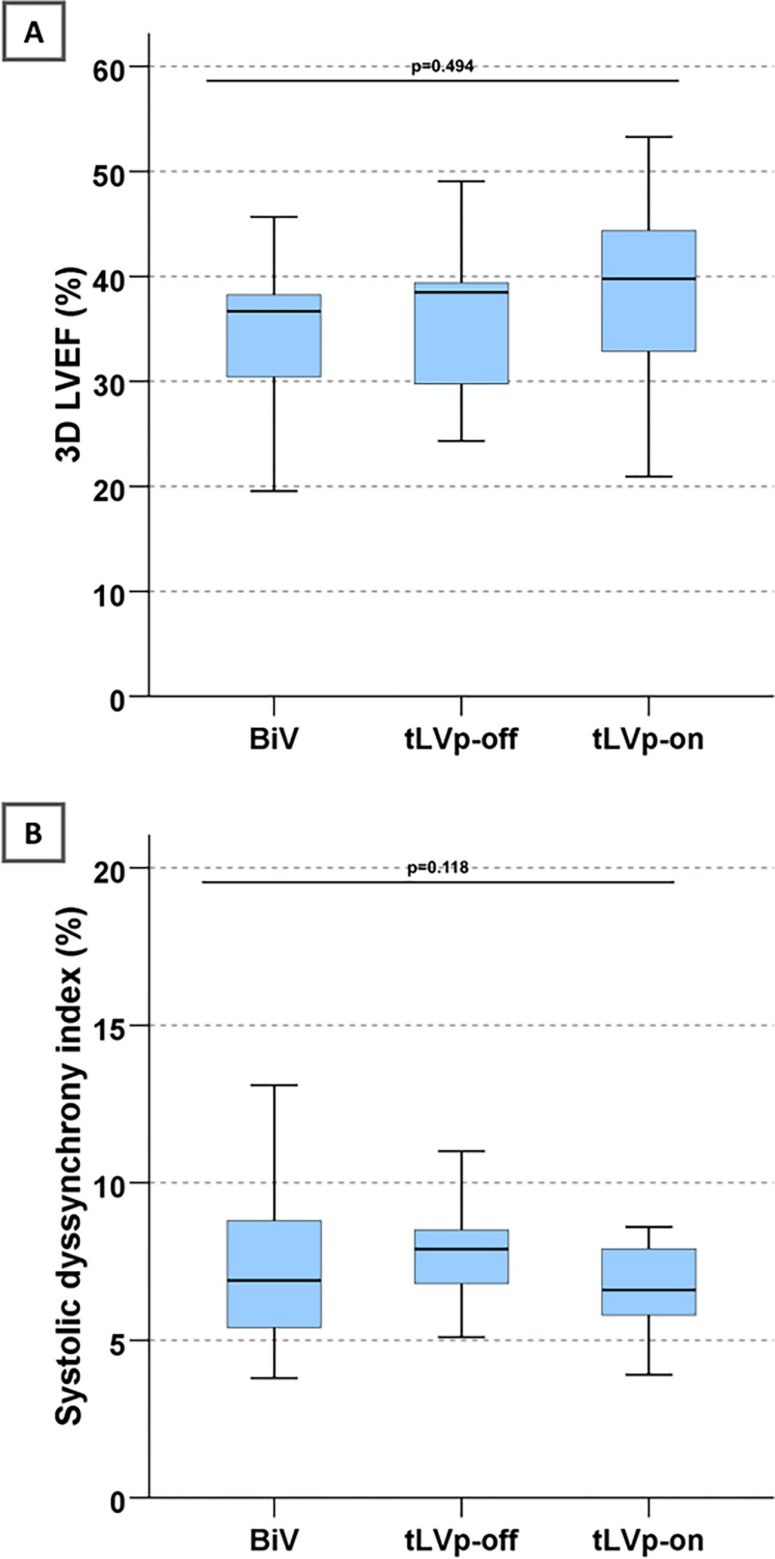
Echocardiographic (echo) markers in the different CRT modes. (**A**) 3D LVEF (echo); (**B**) Systolic dyssynchrony index (SDI; echo). (3D - 3-dimensional, BiV–biventricular pacing, LV—left ventricle, LVEF–left ventricular ejection fraction, tLVp—triggered left ventricular pacing).

Ejection fraction analyzed by 3D volumetry showed no significant change between the three different CRT modes (p = 0.494; Fig 3). In addition to evaluation of 3D ejection fraction we evaluated LV dyssynchrony, using 3D full volume data sets as described above. Systolic Dyssynchrony Index did not show significant differences between the three different settings (p = 0.118; Fig 3).

**Fig 4:**
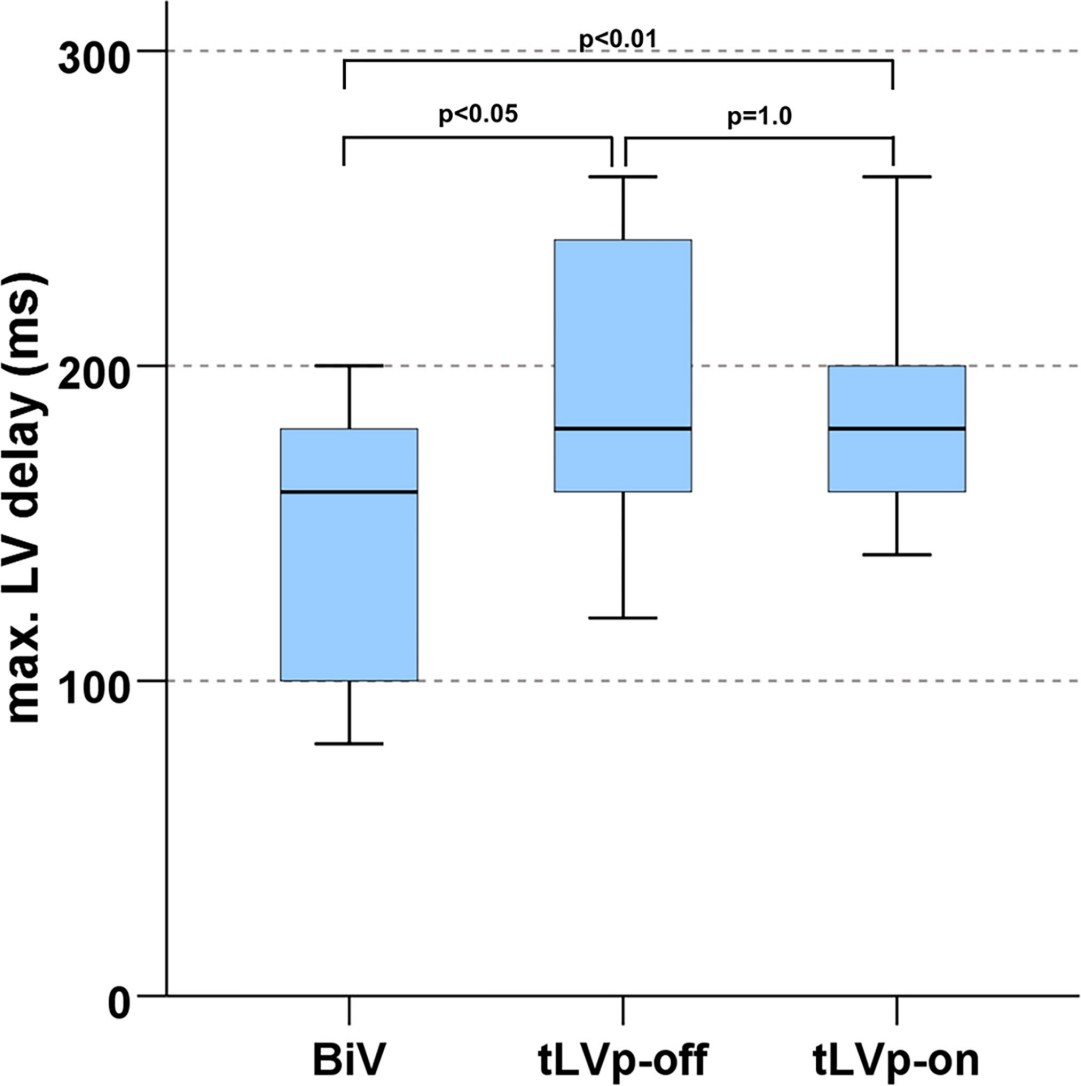
LV delay, measured by 2D strain (echo) in the different CRT modes. **Max.** (BiV–biventricular pacing, LV—left ventricle, LVEF–left ventricular ejection fraction, tLVp—triggered left ventricular pacing).

Besides 3D SDI we calculated LV dyssynchrony using 2D LS analysis as maximum delay of two opposite segments in an apical view as described above. Left ventricular dyssynchrony reached significantly higher values during tLVp-off compared to optimal BiV-Stim settings (p = 0.011). Remarkably, tLVp-algorithms (tLVp-on) did not improve mechanical LV dyssynchrony (p = 1.0; Fig 4).

**Fig 5:**
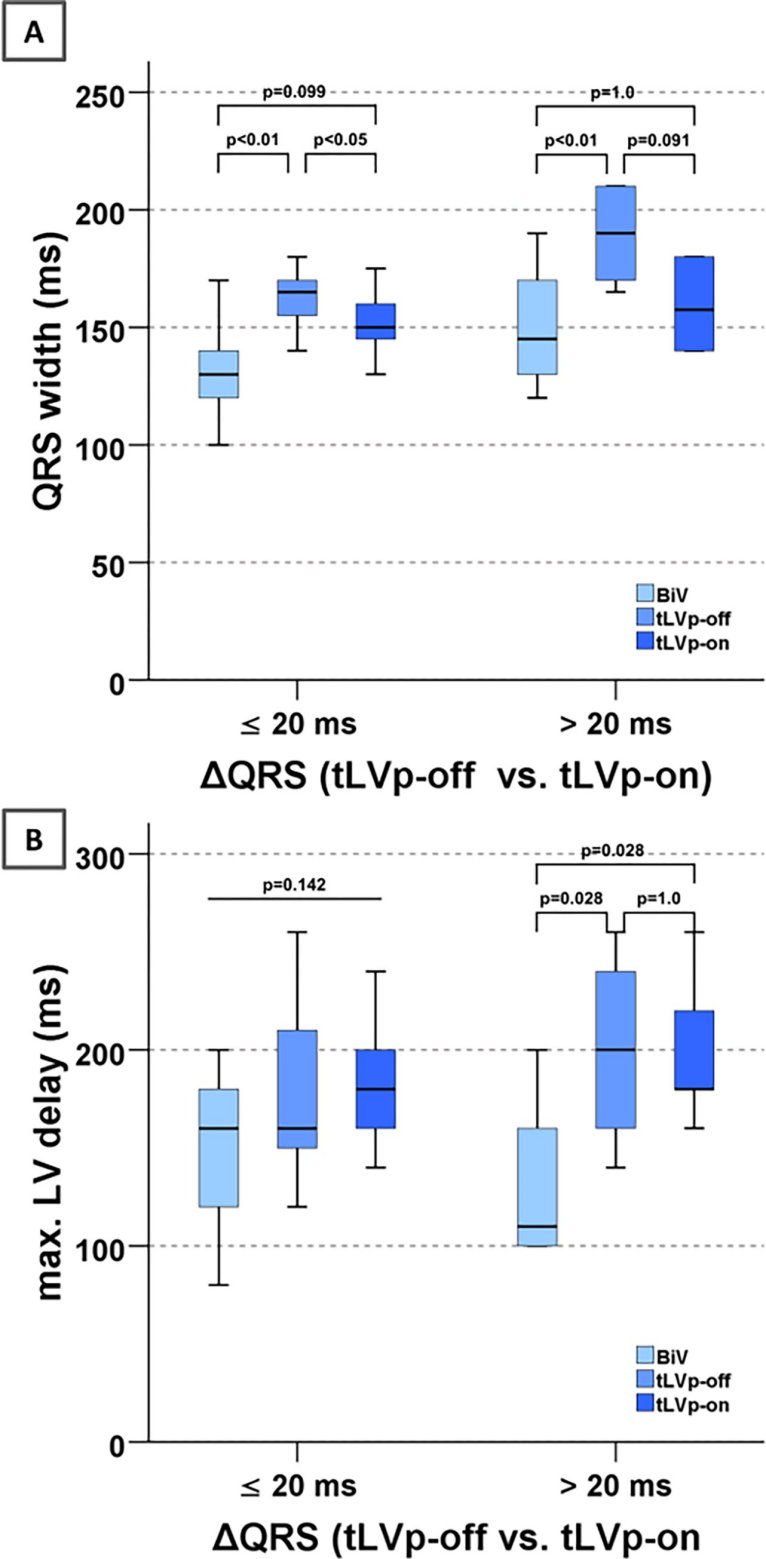
(A) QRS width; (B) Max. LV delay depending on the amount of QRS shortening by tLVp with regard to intrinsic QRS complex (BiV–biventricular pacing, LV—left ventricle, tLVp—triggered left ventricular pacing); n = 9 for ≤20ms, n = 8 for >20ms.

In order to examine the significance of the tLVp algorithm more closely, a distinction was made depending on the amount of QRS shortening by the tLVp algorithm compared to the intrinsic QRS complex (tLVp-off). Even with a relevant shortening (> median of 20 ms), there was a significant difference between the biventricular stimulation and the two other modes (i.e., tLVp-off and tLVp-on; p<0.05) with regard to the maximal mechanical delay that was detected by 2D echocardiography. There was no significant difference regarding the mechanic delay between tLVp-off and tLVp-on (Fig 5). All patients in the subgroup with and QRS shortening >20 ms had a typical LBBB with an intrinsic QRS width > 150 ms (mean QRS width: 174±22 ms).

Furthermore, we also measured the delay between RV- and LV-sense (mean delay 106±47 ms) as well as the delay between Q-wave and LV sense (mean delay 125±47 ms) and compared these data to the echocardiographic response to the different CRT-settings. There was no correlation between the delay between RV- and LV-sense or the delay between Q-wave and LV sense and the echocardiographic response to the different CRT-settings.

An overview of the parameters, depending on the shortening of the QRS complex by tLVp, is shown in Table 2. There was only a significant difference for the intrinsic QRS complex (p<0.05).

**Table 2:** Electrocardiographic and echocardiographic markers depending on the amount of QRS shortening by tLVp with regard to intrinsic QRS complex.

Δ QRS			
(tLVp-off vs. tLVp-on)			
	≤ 20 ms (median)	> 20 ms (median)	p-value
QRS width (ms)			
BiV	130 [120; 140]	145 [130; 170]	0.256
tLVp-off	165 [150; 170]	190 [170; 210]	0.037
tLVp-on	150 [140; 160]	158 [140; 180]	0.733
Max. LV delay (ms)			
BiV	160 [100; 180]	110 [100; 160]	0.404
tLVp-off	160 [140; 240]	200 [160; 240]	0.525
tLVp-on	180 [160; 200]	180 [180; 220]	0.591
3D LVEF (%)			
BiV	37 [30; 39]	36 [24; 38]	0.591
tLVp-off	38 [27; 42]	35 [30; 39]	0.961
tLVp-on	40 [33; 46]	38 [33; 44]	1.000
SDI (%)			
BiV	6.8 [5.4; 8.8]	8.3 [5.1; 11.2]	0.462
tLVp-off	7.2 [5.9; 8.5]	8.1 [6.8; 11]	0.525
tLVp-on	6.7 [5.8; 7.9]	6.3 [5.4; 8.6]	0.808

(3D - 3-dimensional, BiV–biventricular pacing, LV—left ventricle, LVEF–left ventricular ejection fraction, SDI–systolic dyssynchrony index, tLVp—triggered left ventricular pacing); n = 9 for ≤20ms, n = 8 for >20ms.

## Discussion

CRT plays an essential role in the therapy of patients suffering from chronic HFrEF and broad QRS complex. However, in order to achieve beneficial effects, the presence of a broad LBBB is of crucial relevance [7, 25–27]. Beyond that, a high percentage of BiV pacing is essential for clinical response but can be challenging to achieve. For example, atrial fibrillation or other atrial tachycardias as well as PVCs may diminish BiV pacing [8–11]. A high BiV pacing rate might be achieved by pharmacological treatments like beta-blockers, digitalis or amiodarone [28]. Furthermore, electrophysiological procedures such as pulmonary vein isolation, atrioventricular junction ablation (AVJA) and ablation of atrial and ventricular tachycardia or ectopic beats are well-established strategies to ensure a high BiV pacing rate [29–34]. In addition, CRT device manufacturer designed algorithms to ensure BiV pacing, which however increases battery consumption and their effect on clinical outcome has not been proven yet. We therefore investigated the effects of these tLVp-algorithms on electrocardiographic and echocardiographic parameters.

The main findings of the current study are:

1. Independent from QRS duration reduction by tLVp, only BiV pacing seems to improve LV synchrony compared to intrinsic AV conduction, while tLVp fails to improve LV synchrony.
2. When using echocardiography for studying LV synchrony, 2D speckle tracking analyses appear to be more sensitive and suitable than 3D studies and should be preferred in this context.

The results of our observation complement previous study results [35]. The mechanism underlying effective resynchronization therapy using CRT is ideal fusion of right and left ventricular pacing. This must be based on optimal timing, which is achieved either by near-simultaneous pacing, or sometimes premature pacing via the left ventricular lead [36], which is impossible to achieve with tLVp. In tLVp, stimulation via the left ventricular lead and subsequent myocardial activation can only occur with a delay after the beginning of depolarization of the septum or the RV apex has occurred. This probably translates into a less coordinated activation pattern. According to our data, this altered activation pattern leads to a mechanically less coordinated process than classic biventricular stimulation.

With regard to the positive effects of long-term biventricular stimulation, especially on remodeling and ejection fraction, we hypothesize that this cannot be achieved with a high proportion of tLVp. However, this cannot be deduced from our data due to the short term character of our study analyzing only the acute effects and would have to be examined in a separate prospective study.

Our results derived from echocardiographic examinations showed a significant improvement of LV synchronicity during BiV stimulation. However, tLVp algorithms did not significantly improve LV synchronicity compared to physiologic AV nodal conduction. Strain analyses using speckle tracking were chosen to detect these potential delays in myocardial contraction, because of their high temporal resolution and the ability of track sections in any direction within the image plane [37]. No significant differences in LV synchronicity could be detected by 3D volumetry or 3D dyssynchrony index. This observation goes in line with former studies and can be explained by the lower frame rate of 3D echocardiographic acquisitions of current echocardiography machines. Furthermore, the five-minute equilibration interval, which was provided between programming modes, might be too short to enable significant changes of the ejection fraction. However, Witt et al. reported a significantly improved 2D LVEF during BiV compared to intrinsic conduction and tLVp after an equilibrium period of only two minutes. The different findings may be caused by a somewhat different patient cohort, since patients enrolled by Witt et al. presented with a relevant higher LVEF during BiV (47% vs. 37% in our study) [35].

In contrast to 3D volumetry and 3D dyssynchrony index, 2D longitudinal strain analysis revealed that tLVp-algorithms did not improve LV dyssynchrony, whereas BiV pacing did significantly reduce LV dyssynchrony, showing that tLVp pacing is inferior to BiV pacing regarding the treatment of dyssynchrony.

Therefore, improving the BiV pacing rate by pharmacological and ablation therapies should be preferred over a relevant percentage of tLVp pacing. However, in some patients these therapeutic options are limited, for example due to comorbidities, allergic reactions or the volition of the patient.

### Study limitations

A limitation of this study is the small number of included patients. This is in part a result of the study design. By choosing complex echocardiographic parameters to detect LV dyssynchrony, only patients with sufficient acoustic window could be included in the study. In a cohort of patients suffering from HFrEF a significant portion presents with previous cardiac surgery reducing optimal acoustic windows for echocardiography and advanced echocardiographic analyses, especially those based on 3D echocardiograms rely on echocardiograms of outstanding quality. Furthermore, an intrinsic atrioventricular nodal conduction with a PR interval of less than 200ms was required due to the study design. As many heart failure patients require CRT due to bradyarrhythmia with high RV pacing rates, these patients frequently could not be included [38, 39]. Especially analysis of pacing rates with and without tLVp-algorithm were challenging, as only devices of one manufacturer (Medtronic) provides the possibility to differentiate between pacing rates with and without tLVp-algorithm. Therefore, our patient count concerning this variable remained too small to draw any further conclusions. However, earlier reports on tLVp-algorithms were based on similar small patient cohorts [35, 40] and our patient cohort was big enough to form two groups depending on changes QRS width to further analyze the echocardiographic findings. Beyond that, a strength of our patient cohort lies in the variety of different cardiomyopathies and devices from different manufacturers representing a real-world collective.

## Conclusions

In conclusion our data show that tLVp-algorithms did enhance cardiac synchronicity compared to intrinsic atrioventricular nodal conduction without tLVp. However, compared to BiV pacing, the effect of tLVp was inferior regarding treatment of LV dyssynchrony. Therefore, BiV pacing should preferably be reached by pharmacological treatment and electrophysiological ablation procedures. If high BiV pacing are not achievable by these treatment options, tLVp-algorithms might provide some beneficial effects.
